# Clinically reported covert cerebrovascular disease and risk of neurological disease: a whole-population cohort of 367 988 people using natural language processing

**DOI:** 10.1136/jnnp-2025-337689

**Published:** 2026-03-13

**Authors:** Matthew Henry Iveson, Mome Mukherjee, Emma M Davidson, Huayu Zhang, Laura Sherlock, Emily L Ball, Grant Mair, Alice Hosking, Heather Whalley, Michael T C Poon, Joanna M Wardlaw, David M Kent, Richard Tobin, Claire Grover, Beatrice Alex, William Whiteley

**Affiliations:** 1 Institute for Neuroscience and Cardiovascular Research, The University of Edinburgh, Edinburgh, UK; 2 Nuffield Department of Primary Care Health Sciences, University of Oxford, Oxford, UK; 3 Kellogg College, University of Oxford, Oxford, UK; 4 Centre for Clinical Informatics, The University of Edinburgh Usher Institute of Population Health Sciences and Informatics, Edinburgh, UK; 5 Centre for Medical Informatics, The University of Edinburgh Usher Institute of Population Health Sciences and Informatics, Edinburgh, UK; 6 UK Dementia Research Institute, Edinburgh, UK; 7 Graduate School of Biomedical Sciences, Tufts University, Medford, Massachusetts, USA; 8 School of Informatics, The University of Edinburgh, Edinburgh, UK; 9 School of Literatures, Languages and Cultures, The University of Edinburgh, Edinburgh, UK; 10 Advanced Care Research Centre, The University of Edinburgh, Edinburgh, UK; 11 British Heart Foundation Data Science Centre, HDR UK, London, UK

**Keywords:** STROKE, DEMENTIA, CEREBROVASCULAR DISEASE, EPIDEMIOLOGY

## Abstract

**Background:**

The relevance of covert cerebrovascular disease (CCD) in practice is uncertain, partly because estimation of risk in whole clinical populations is difficult. Studies have had success extracting CCD from clinical text using natural language processing (NLP), though they have been limited to specific CCD phenotypes. Here, we used NLP to measure multiple clinically-reported CCD phenotypes in a large clinical cohort and estimated subsequent disease risk in health record data.

**Methods:**

From all people with brain imaging in Scotland (2010–2018), we selected people with no prior hospitalisation for neurological disease (n=367 988). NLP of imaging reports identified: white matter hypoattenuation or hyperintensities (WMH), lacunes, cortical infarcts and cerebral atrophy. Adjusted HRs (aHRs) were estimated between each phenotype and stroke, dementia and Parkinson’s disease (conditions previously associated with CCD), epilepsy and colorectal cancer (control conditions).

**Results:**

For each phenotype, the aHR of stroke was WMH 1.4 (95% CI 1.3–1.4), lacunes 1.6 (1.5–1.6), cortical infarct 1.8 (1.7–1.9) and cerebral atrophy 1.1 (1.0–1.1). The aHR of dementia was WMH 1.3 (1.3–1.3), lacunes 1.0 (0.9–1.0), cortical infarct 1.1 (1.1–1.2) and cerebral atrophy 1.7 (1.7–1.8). The aHR of Parkinson’s disease was WMH 1.1 (1.0–1.2), lacunes 1.1 (0.9–1.2), cortical infarct 0.7 (0.6–0.9) and cerebral atrophy 1.4 (1.3–1.5). The aHRs between CCD phenotypes and epilepsy and colorectal cancer were around the null.

**Conclusion:**

CCD and atrophy have implications for future disease risk and can be identified at scale using NLP of clinical reports. Prevention of neurological disease in people with CCD should be a priority for healthcare policy makers.

**WHAT IS ALREADY KNOWN ON THIS TOPIC**

- Systematic reviews of MRI in cohort studies show that people with asymptomatic, covert cerebrovascular disease (CCD) have an increased risk of stroke, dementia and Parkinson’s disease.

**WHAT THIS STUDY ADDS**

- This study used a validated natural language processing (NLP) algorithm to identify three distinct CCD phenotypes and cerebral atrophy from both MRI and CT imaging reports generated during routine healthcare across a whole population. We demonstrate a higher risk of dementia (particularly Alzheimer’s disease) in people with cerebral atrophy and a higher risk of stroke in people with cortical infarcts, but no significant associations with an age-associated control outcome (colorectal cancer), supporting a causal relationship.

**HOW THIS STUDY MIGHT AFFECT RESEARCH, PRACTICE OR POLICY**

- We demonstrate that NLP can identify important CCD and cerebral atrophy phenotypes that are otherwise difficult to identify in electronic health records. This allows research at scale into imaging-based biomarkers of dementia and stroke.

## Introduction

 Covert cerebrovascular disease (CCD) is a common incidental finding after brain imaging. CCD imaging phenotypes include subcortical and periventricular white matter hypoattenuation and hyperintensities (WMH), small subcortical (lacunar) infarcts and large artery atherothrombo- or cardio-embolic (cortical) infarcts in people who have never reported symptoms of stroke or transient ischaemic attack (TIA). Systematic reviews of cohorts screened by MRI show that CCD prevalence is much higher than symptomatic stroke,^1^,^3^ and that findings of CCD are independently associated with stroke and dementia in population-based cohorts.^4^ However, these findings may not generalise to clinical practice because participants in cohort studies are not representative of people seeking healthcare^5^ and because neuroimaging reports generated during research are more standardised than clinical reports. Furthermore, while most studies identify CCD with MRI, CT is more commonly used in clinical practice, particularly for those with minor symptoms for whom there is clinical uncertainty. Therefore, the clinical relevance of clinically reported CCD is often uncertain.

CCD does not have a diagnostic code in common classifications (eg, International Classification of Diseases (ICD)−10); hence, it is difficult to study in populations representative of clinical practice. While new classifications such as ICD-11 may improve this, they are not widely adopted across electronic health records. Natural language processing (NLP) can help to identify hard-to-find phenotypes in medical notes and clinical reports. We have developed and validated a rules-based NLP algorithm (EdIE-R) with excellent recall and precision to identify all available brain imaging phenotypes from brain imaging radiology reports.^6^ This algorithm performed with high accuracy, compared with a reference standard of expert reads of brain imaging reports or brain images.^6^,^9^ Therefore, NLP could reveal CCD and atrophy phenotypes in whole populations of patients who have had brain imaging during routine healthcare, allowing estimation of risk in clinical populations. Previous studies have shown that NLP-derived indicators of brain infarcts and white matter disease are associated with stroke and dementia risk among older adults.^10 11^

In this study, we used NLP to identify clinically reported CCD and cerebral atrophy on CT and MRI brain imaging reports from all routine healthcare in the Scottish population for up to 12 years after index imaging. We estimated the absolute risks and association of clinically reported CCD and atrophy with incident stroke, dementia and Parkinson’s disease and with epilepsy (a brain-based control condition not directly related to CCD) and colorectal cancer (a non-brain control condition).

## Methods

### Study design and participants

We used deidentified individual-level data, accessed through the National Health Service (NHS) Scotland’s National Safe Haven provided by Public Health Scotland (PHS). PHS deterministically linked general hospital inpatient records (Scottish morbidity record (SMR) 01), mental health inpatient records (SMR 04), community prescribing (Prescribing Information System (PIS)), cancer registrations (SMR 06), NRS death records and brain imaging reports from Scottish Medical Imaging with the community health index number, a unique patient identifier, between 1 January 2008 and 31 December 2020.^12^

**Figure 1:**
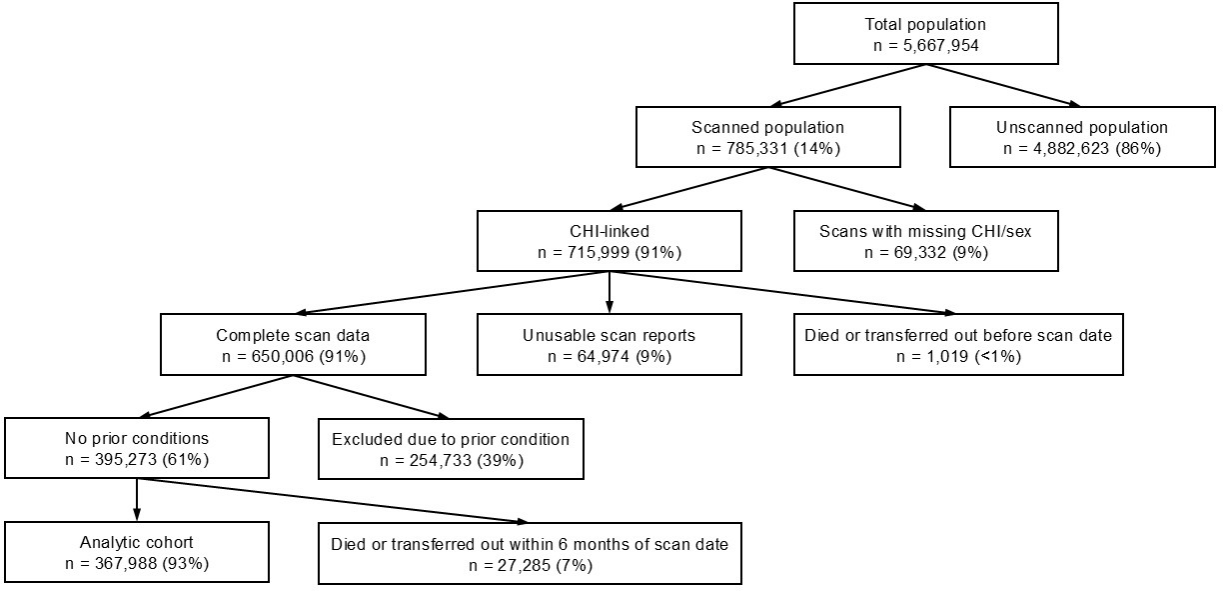
Flow diagram showing cohort selection from the whole population of Scotland who interacted with the Scottish NHS (n=5 667 954) to the analytic cohort (n=367 988). Percentages indicate the proportion of the node above. CHI, Community Health Index.

We extracted data from the whole population of Scotland with at least one NHS Scotland hospitalisation, outpatient or prescribing record (5 667 954 people). We selected all 785 331 people with a brain MRI or CT scan between January 2010 and August 2018. We excluded people with missing demographic data, an imaging date that conflicted with emigration or death, reports from research studies (eg, clinical trials), reports with no usable information and reports of non-brain imaging. We excluded people with records of stroke of any type, dementia, epilepsy, colorectal cancer, multiple sclerosis, Parkinson’s disease, TIA, subarachnoid haemorrhage, subdural haemorrhage and extradural haemorrhage, prior to the date of scan or 6 months after the date of scan (online supplemental material). We then removed individuals who died or transferred out within 6 months after the date of scan. The process of selecting the analytic cohort is shown in figure 1.

### Study definitions

CCD and atrophy imaging phenotypes were white matter hypoattenuation (on CT) or hyperintensities (on MRI) (WMH), lacunes (small subcortical infarcts), cortical infarcts and cerebral atrophy. Cerebral atrophy was included because it is a common cofinding of CCD and has potentially important disease associations. Rules-based NLP identified CCD or atrophy on the first available MRI or CT brain imaging report for each participant (https://www.ltg.ed.ac.uk/software/EdIE-R/). Reports were created during the course of routine healthcare.^13^ The tool has been shown to perform well (F1 scores generally above 90%) against expert reads of Scottish brain imaging reports and against an expert-independent rater reading underlying images.^6 9^ Additionally, we created a variable summing the number of CCD and atrophy phenotypes present at the first brain image.

We defined outcomes as the first recorded date of any stroke (and subtypes ischaemic stroke, intracerebral haemorrhage and uncertain stroke); all dementia (and subtypes Alzheimer’s dementia, vascular dementia and unspecified or rare dementia); Parkinson’s disease; epilepsy and colorectal cancer. Epilepsy and colorectal cancer were included as brain-based and non-brain negative control outcomes, respectively. Outcomes were defined with established ICD10 coding and British National Formulary medication lists (online supplemental material) taken from the Health Data Research UK Phenotype Library (https://phenotypes.healthdatagateway.org/) with SMR01, SMR04, SMR06, PIS and national death records.

Covariates available from electronic health records included age (in years) at first-available imaging report date, split into 5-year bands, sex, area-level measures of deprivation at first brain imaging (Scottish Index of Multiple Deprivation decile (2012 classification),^14^ Urban-Rural Classification (eightfold 2011 classification)^15^ and Health Board of scan (a proxy for region)). Ethnicity data were not available for the cohort.

We used all data sources to define comorbidities prior to first imaging: myocardial infarction (MI), angina, peripheral vascular disease, renal impairment, heart failure, non-colorectal cancer and diabetes (online supplemental material). We measured healthcare usage in the year prior to first scan with the number of hospital admissions, the number of unique medicines dispensed, the number of statin prescriptions dispensed and the number of antihypertensive prescriptions dispensed.

Follow-up started from 6 months after the date of the first brain imaging report (index date), to allow time for diagnosis made with or because of the first brain imaging to appear in records. For each outcome separately, survival time was estimated to an event, or right-censored on death, migration out of Scotland or study end (31 December 2020). We report 1-year, 5-year and overall cumulative incidence with the Kaplan-Meier method for each outcome by scan phenotypes (WMH, lacunes, cortical infarcts and cerebral atrophy) and survival estimates relative to the wider Scottish population (the analytic cohort plus the unscanned population alive on 01 July 2010; n=4 637 231). Survival analyses were conducted with Cox proportional hazards models, and the proportional hazards assumption was tested statistically and visually with Schoenfeld residual plots. The variables ‘scan modality’ and the Scottish Health Board violated this assumption and were included as stratification variables. For each outcome (and its subtypes) and for each CCD and atrophy phenotype, we estimated maximally adjusted HRs (aHRs), adjusting for other phenotypes, age, sex, deprivation, rurality, comorbidities and healthcare usage measured by prescriptions and admissions in the year prior to the scan. In further analyses, we examined the aHRs for the number of CCD and atrophy phenotypes at scan (max 4). Full model estimates, including univariate HRs, are shown in the online supplemental material.

Sensitivity analyses for each outcome (and subtype) tested whether diagnosis preceded scan by excluding the first 1 and 5 years of follow-up. While the first 6 months after the scan are excluded in the primary analyses, excluding larger time periods provides a stricter test of potential asymptomatic time, particularly for conditions with more insidious onset. Subgroup analyses tested aHR by age, sex and brain imaging type.

Analyses were conducted within the NHS Scotland National Safe Haven, using R (V.4.3.2)^16^ and R Studio (V.2023.12.0)^17^ with the package ‘survival’.^18^ Missing data were not imputed. P values are reported uncorrected for multiple comparisons.

The study was approved by the NRES Committee Northwest—Greater Manchester East Ethics Committee (15/NW/0719) and the Public Benefit and Privacy Panel for Health and Social Care (1516–0219) of NHS Scotland.

## Results

Of 5.67 million people in Scotland between 2008 and 2018, 0.65 million (11%) had at least one brain imaging report, of whom 0.36 million (57%) had no recorded history of neurological disease and were alive 6 months after the scan. Of these 0.36 million, most (54%) were over 55 years old; 22% had a report of WMH, 8% of a lacune, 2% of a cortical infarct, 22% of cerebral atrophy, and 65% had none of these scan phenotypes. There was a total of 2.4 million person-years of follow-up time, with a median follow-up time of people with CCD or atrophy of 5.0 years and of scanned individuals without CCD or atrophy of 7.0 years (online supplemental material).

Compared with people without, those with CCD or atrophy were older (mean 73.4 vs 48.2 years); had more admissions with MI (13.2% vs 3.8%), renal impairment (9.2% vs 1.9%), heart failure (5.1% vs 1.0%), cancer (10.7% vs 5.4%) and diabetes (9.7% vs 3.3%) and had a greater number of hospitalisations (mean 1.1 vs 0.7) and prescribed medicines (3.1 vs 1.6) in the year prior to the scan (table 1). Compared with the Scottish population (with or without a brain imaging), people who had brain imaging were older, had more vascular and cancer comorbidities and had fewer antihypertensive and statin dispensing events in the year prior to the scan.

**Table 1:** Demographic characteristics of those with and without clinically reported covert cerebrovascular (CCD) or cerebral atrophy phenotype and of the whole Scottish population (analytic cohort plus unscanned individuals alive at 01 July 2010)

	Analytic cohort			Scottish population
	Covert cerebrovascular disease or cerebral atrophy n=129 199	No covert cerebrovascular disease or cerebral atrophy n=238 789	All n=367 988	n=4 637 231
Sex				
Female	69 246 (53.6%)	129 957 (54.4%)	199 203 (54.1%)	2 457 323 (53.0%)
Age (years)				
Mean (SD)	73.4 (12.6)	48.2 (17.1)	57.0 (19.8)	40.9 (22.5)
Age group				
≤25	324 (0.3%)	26 526 (11.1%)	26 850 (7.3%)	613 158 (13.2%)
26–35	896 (0.7%)	38 404 (16.0%)	39 300 (10.7%)	616 072 (13.3%)
36–45	2675 (2.1%)	41 770 (17.5%)	44 445 (12.1%)	684 817 (14.8%)
46–55	8395 (6.5%)	49 818 (20.8%)	58 213 (15.8%)	703 974 (15.2%)
56–65	17 788 (13.8%)	40 537 (17.0%)	58 325 (15.9%)	589 770 (12.7%)
66–75	34 841 (27.0%)	26 853 (11.2%)	61 694 (16.8%)	400 205 (8.6%)
76–85	44 257 (34.2%)	12 051 (5.1%)	56 308 (15.3%)	227 668 (4.9%)
86–95	18 958 (14.7%)	2711 (1.2%)	21 669 (5.9%)	77 073 (1.7%)
≥96	1065 (0.8%)	119 (<0.1%)	1184 (0.3%)	6638 (0.1%)
Scan modality				
CT	105 977 (82.0%)	167 623 (70.2%)	273 600 (74.4%)	–
MRI	23 222 (18.0%)	71 166 (29.8%)	94 388 (25.6%)	–
SIMD* quintile				
1 most deprived	29 800 (23.1%)	58 365 (24.4%)	88 165 (24.0%)	–
2	26 887 (20.8%)	48 793 (20.4%)	75 680 (20.6%)	–
3	23 863 (18.5%)	44 398 (18.6%)	68 261 (18.5%)	–
4	23 043 (17.8%)	41 734 (17.5%)	64 777 (17.6%)	–
5 least deprived	23 204 (18.0%)	38 449 (16.1%)	61 653 (16.8%)	–
Missing	2402 (1.8%)	7050 (3.0%)	9452 (2.5%)	–
Urban–rural†				
1 large urban	53 271 (41.2%)	96 467 (40.4%)	149 738 (40.7%)	–
2	38 468 (29.8%)	70 018 (29.3%)	108 486 (29.5%)	–
3	10 649 (8.3%)	18 752 (7.9%)	29 401 (8.0%)	–
4	3100 (2.4%)	4878 (2.0%)	7978 (2.2%)	–
5	1127 (0.9%)	2210 (0.9%)	3337 (0.9%)	–
6	12 831 (9.9%)	24 106 (10.1%)	36 937 (10.0%)	–
7	3669 (2.8%)	6427 (2.7%)	10 096 (2.7%)	–
8 very remote rural	2928 (2.3%)	5443 (2.3%)	8371 (2.3%)	–
Missing	3156 (2.4%)	10 488 (4.4%)	13 644 (3.7%)	–
Comorbidities				
MI, angina or peripheral vascular disease	17 085 (13.2%)	8978 (3.8%)	26 063 (7.1%)	81 427 (1.8%)
Renal impairment	11 922 (9.2%)	4555 (1.9%)	16 477 (4.5%)	35 510 (0.8%)
Heart failure	6639 (5.1%)	2283 (1.0%)	8922 (3.2%)	25 586 (0.6%)
Any cancer	13 886 (10.7%)	13 000 (5.4%)	26 886 (7.3%)	84 033 (1.8%)
Diabetes	12 511 (9.7%)	7916 (3.3%)	20 427 (5.6%)	58 600 (1.3%)
Care in year before scan				
Hospital admissions (mean, SD)	1.1 (2.4)	0.7 (1.9)	0.8 (2.1)	0.8 (1.8)
Unique medicines (mean, SD)	3.1 (2.7)	1.6 (2.1)	2.1 (2.4)	2.8 (2.5)
Antihypertensive dispensing events (mean, SD)	2.3 (3.6)	0.8 (2.3)	1.3 (2.9)	3.7 (6.9)
Statin dispensing events (mean, SD)	1.0 (1.6)	0.3 (1.0)	0.6 (1.3)	1.5 (3.1)

* 2012.

† 2011–2012.

MI, myocardial infarction; MRI, magnetic resonance imaging; SIMD, Scottish Index of Multiple Deprivation.

**Figure 2:**
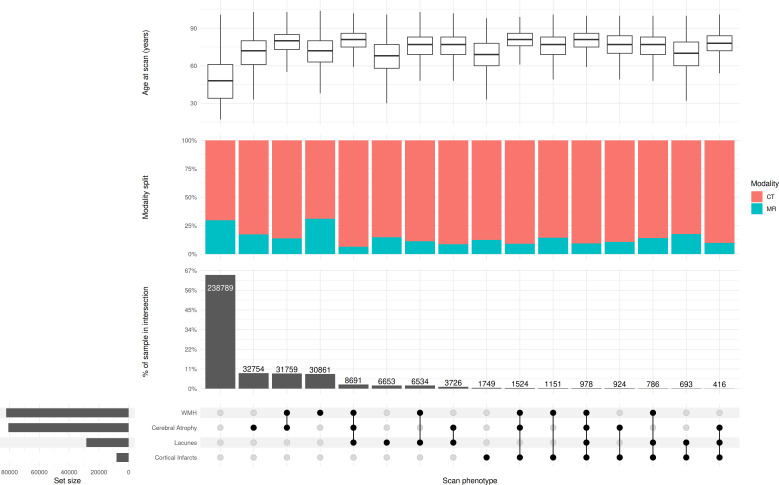
Co-occurrence of covert cerebrovascular disease phenotypes, with summary of imaging modality and age at imaging per intersection. Box and whisker plots represent range, IQR and median. WMH, white matter hypoattenuation/hyperintensities.

The five most frequent combinations of CCD and atrophy phenotypes were cerebral atrophy alone; WMH and cerebral atrophy; WMH alone; WMH, cerebral atrophy and lacunes; and lacunes. The proportion of people who had an MRI rather than a CT was higher in people with no phenotype and for WMH only, although for all phenotype combinations, most brain imaging was performed with CT (figure 2).

The incidence rate, absolute risk difference and relative risk (RR) of outcomes between people with a brain image report and the wider Scottish population are shown in the online supplemental material. Incidence was proportionally higher in those with a brain image report than in the wider population for all outcomes except colorectal cancer, though RRs were lower with increasing age (online supplemental material). At 1 and 5 years, people with a CCD or atrophy phenotype were more likely than the wider Scottish population to experience a stroke (1 year: RR 6.4, 95% CI 6.1 to 6.7; 5 years: RR 30.3, 95% CI 29.6 to 31.0), dementia (RR 5.1, 95% CI 5.0 to 5.2; RR 16.8, 95% CI 16.6 to 17.0), Parkinson’s disease (RR 3.5, 95% CI 3.3 to 3.8; RR 18.1, 95% CI 17.4 to 18.9) and epilepsy (RR 1.9, 95% CI 1.8 to 1.9; RR 7.9, 95% CI 7.8 to 7.9) but not colorectal cancer (RR 1.1, 95% CI 0.9 to 1.2; RR 5.1, 95% CI 4.9 to 5.3). At 1 year, the cumulative incidence (accounting for the competing risk of death) of stroke and dementia was similar and low among those with WMH, lacunes, cortical infarcts, cerebral atrophy or no scan phenotype (around 2% and 9%; table 2). At 5 years, cumulative stroke incidence was highest among those with cortical infarcts (11%), followed by lacunes (9%), WMH (7%) and cerebral atrophy (6%), while dementia incidence was highest among those with cerebral atrophy (26%), followed by WMH (23%), cortical infarcts (22%) and lacunes (22%).

**Table 2:** 1-year and 5-year cumulative incidence (%) for each scan phenotype and outcome, accounting for competing risk of death

Outcome	WMH	Lacunes	Cortical infarcts	Cerebral atrophy	None	Analytic cohort	Scottish population
1 year							
Stroke	2.0 (2.0–2.1)	2.6 (2.4–2.8)	3.5 (3.1–3.9)	1.8 (1.7–1.9)	0.3 (0.3–0.3)	0.8 (0.8–0.8)	0.1 (0.1–0.1)
Dementia	9.6 (9.4–9.8)	8.4 (8.1–8.7)	9.1 (8.5–9.8)	11.0 (11.0–11.0)	0.8 (0.8–0.9)	3.7 (3.6–3.8)	0.5 (0.5–0.5)
Parkinson’s	0.5 (0.5–0.6)	0.5 (0.5–0.6)	0.3 (0.2–0.5)	0.6 (0.5–0.6)	0.1 (<0.1–0.1)	0.2 (0.2–0.3)	0.1 (<0.1–0.1)
Epilepsy	3.2 (3.1–3.3)	3.2 (3.0–3.4)	3.4 (3.0–3.8)	3.2 (3.0–3.3)	4.1 ((4.0–4.1)	3.9 (3.8–4.0)	2.0 (2.0–2.0)
Colorectal cancer	0.3 (0.3–0.3)	0.3 (0.3–0.4)	0.4 (0.3–0.5)	0.3 (0.3–0.4)	0.1 (<0.1–0.1)	0.2 (0.2–0.2)	0.2 (0.2–0.2)
5 years							
Stroke	6.8 (6.6–7.0)	8.7 (8.4–9.0)	11.0 (10.0–12.0)	6.2 (6.0–6.4)	1.2 (1.1–1.2)	2.9 (2.9–3.0)	0.2 (0.2–0.2)
Dementia	23.0 (23.0–24.0)	22.0 (21.0–22.0)	22.0 (22.0–23.0)	26.0 (25.0–26.0)	2.3 (2.2–2.3)	9.5 (9.4–9.6)	1.0 (1.0–1.0)
Parkinson’s	1.9 (1.8–2.0)	1.9 (1.8–2.1)	1.4 (1.1–1.7)	2.2 (2.1–2.3)	0.4 (0.4–0.4)	1.0 (1.0–1.0)	0.1 (0.1–0.1)
Epilepsy	9.2 (9.0–9.4)	9.2 (8.9–9.6)	9.4 (8.8–10.0)	9.4 (9.2–9.6)	12.0 (12.0–13.0)	12.5 (12.4–12.6)	5.0 (5.0–5.0)
Colorectal cancer	1.0 (0.9–1.1)	1.1 (0.9–1.2)	1.2 (0.9–1.4)	1.1 (1.0–1.2)	0.4 (0.3–0.4)	0.6 (0.6–0.7)	0.5 (0.5–0.5)

WMH, white matter hypoattenuation/hyperintensities.

### Risk of stroke

**Figure 3:**
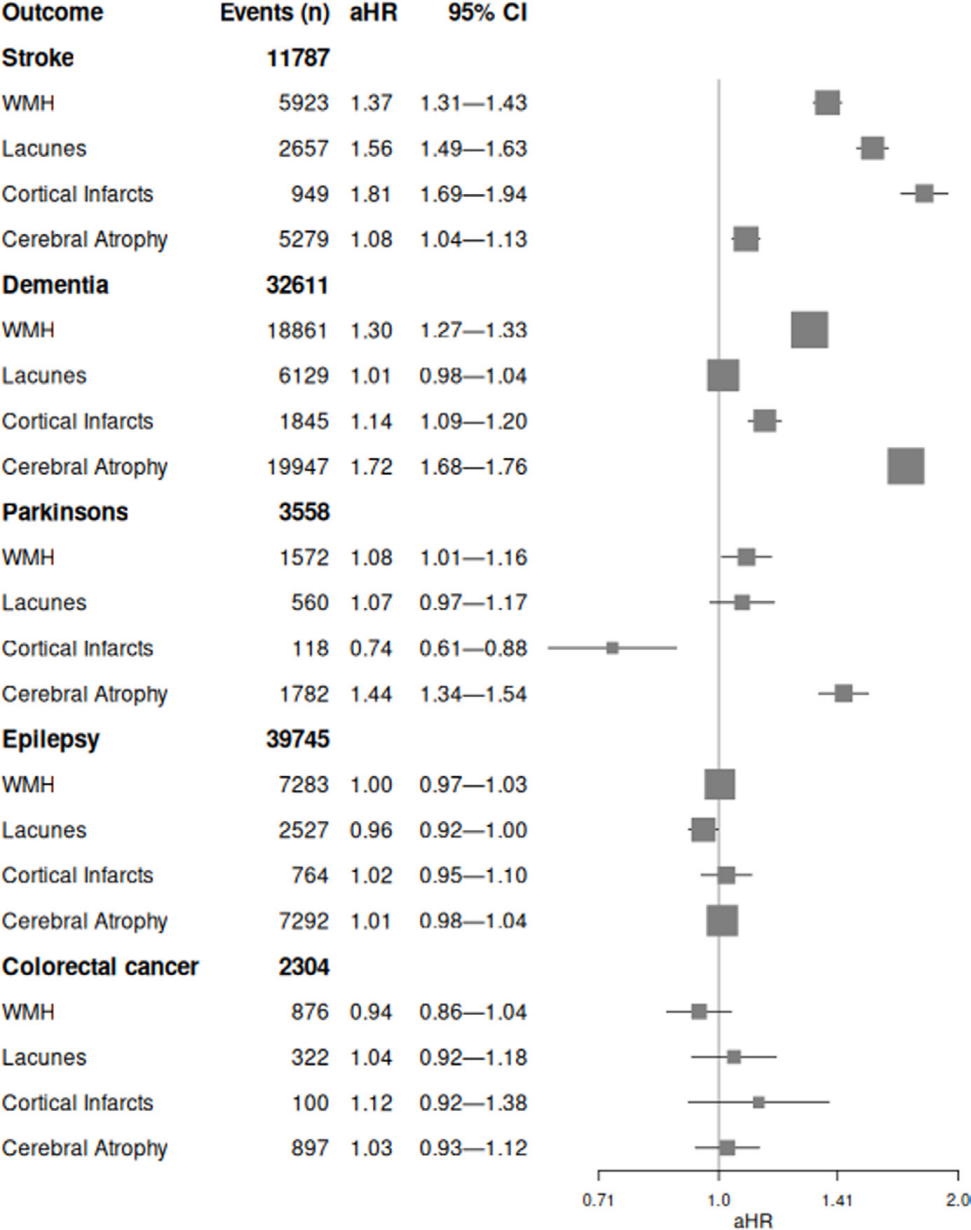
Forest plot showing the event rate and adjusted HR (aHR) and 95% CIs for each exposure (white matter hyper (MRI) or hypointensity (CT) (WMH), lacunes, cortical infarcts and cerebral atrophy) and their association with stroke risk, dementia risk, Parkinson’s risk, epilepsy risk and colorectal cancer risk across follow-up.

**Figure 4:**
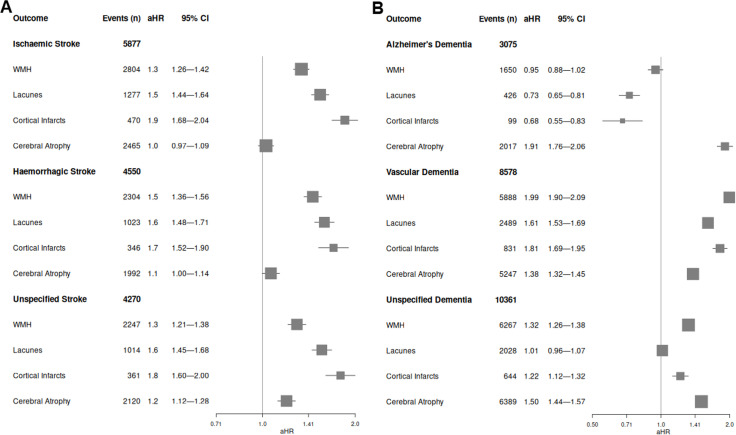
Forest plot showing the event rate and adjusted HR (aHR) and 95% CIs for each exposure (white matter hyper (MRI) or hypointensity (CT) (WMH), lacunes, cortical infarcts and cerebral atrophy) and their association with risk of (A) stroke subtypes and (B) dementia subtypes over follow-up.

Compared with those with no CCD or atrophy phenotype, those with each phenotype (accounting for the presence of other phenotypes) had a higher risk of any stroke over the 12-year follow-up period. The aHRs were, for cortical infarct 1.8 (95% CI 1.7 to 1.9), lacunes 1.6 (95% CI 1.5 to 1.6), WMH 1.4 (95% CI 1.3 to 1.4) and cerebral atrophy 1.1 (95% CI 1.0 to 1.1) (figure 3). The aHR for ischaemic stroke was highest in people with cortical infarct (1.9, 95% CI 1.7 to 2.0) and aHR for intracerebral haemorrhage was highest in people with cortical infarct (1.7, 95% CI 1.5 to 1.9) and lacunes (1.6, 95% CI 1.5 to 1.7). People with cerebral atrophy had an increased risk of unspecified stroke (1.2, 95% CI 1.1 to 1.3) but not ischaemic stroke (1.0, 95% CI 0.9 to 1.1) or haemorrhagic stroke (1.1, 95% CI 0.9 to 1.1) (figure 4). People with more CCD or atrophy phenotypes had a higher aHR for all stroke (1.4, 95% CI 1.3 to 1.4), with the highest aHR (vs those without phenotypes) in those with all four phenotypes (3.0, 95% CI 2.5 to 3.6) (online supplemental material).

### Risk of dementia

Compared with people with no evidence of CCD or atrophy, the aHR for dementia was higher in people with cerebral atrophy (aHR 1.7, 95% CI 1.7 to 1.8), WMH (1.3, 95% CI 1.3 to 1.3) and cortical infarct at scan (1.1, 95% CI 1.1 to 1.2), although not lacunes (1.0, 95% CI 1.0 to 1.0) (figure 3). Of the CCD or atrophy phenotypes, only people with cerebral atrophy at scan had an increased risk of each dementia type: Alzheimer’s disease (1.9, 95% CI 1.8 to 2.1), vascular dementia (1.4, 95% CI 1.3 to 1.4) and unspecified dementia (1.5, 95% CI 1.4 to 1.6). Compared with those without CCD or atrophy phenotypes, people with cortical infarct or lacunes had a higher risk of vascular dementia (cortical infarct: 1.8, 95% CI 1.7 to 2.0; lacunes: 1.6, 95% CI 1.5 to 1.7) but lower incidence of Alzheimer’s disease (cortical infarct: 0.7, 95% CI 0.6 to 0.8; lacunes: 0.7, 95% CI 0.7 to 0.8). Compared with those without WMH, people with WMH had a higher risk of vascular dementia (2.0, 95% CI 1.9 to 2.1) and unspecified dementia (1.3, 95% CI 1.3 to 1.4), but not clearly Alzheimer’s dementia (0.9, 95% CI 0.9 to 1.0) (figure 4). People with more CCD phenotypes had a higher dementia aHR (1.3, 95% CI 1.3 to 1.3), with the highest aHR among those exhibiting all four phenotypes (2.6, 95% CI 2.3 to 2.9).

### Risk of Parkinson’s disease, epilepsy and colorectal cancer

Compared with people with no evidence of CCD or atrophy, the aHR of Parkinson’s disease was lower in people with cortical infarct (aHR 0.7, 95% CI 0.6 to 0.9) and higher in people with cerebral atrophy (1.4, 95% CI 1.3 to 1.5) and WMH (1.1, 95% CI 1.0 to 1.2), but not clearly higher in people with lacunes (1.1, 95% CI 1.0 to 1.2) (figure 3). There was a linear association between more scan phenotypes and Parkinson’s disease risk (1.2, 95% CI 1.1 to 1.2), but risk in those with all four phenotypes did not significantly differ from those with no phenotypes (1.0, 95% CI 0.6 to 1.7). There was a significant association between the presence of lacunes at scan and lower risk of epilepsy, though this association was notably weak (1.0, 95% CI 0.9 to 1.0). None of the other CCD or atrophy phenotypes had a large aHR with risk of epilepsy (aHRs 0.9–1.0) or colorectal cancer (aHRs 0.9–1.1), and all had a p value of >0.05.

### Sensitivity analyses

After excluding the first year and 5 years of follow-up, most estimates were of a similar magnitude and direction to the primary analyses. The exceptions were, after excluding 5 years of follow-up, the aHR for cerebral atrophy with stroke risk was around the null, and aHRs between lacunes and cortical infarcts with dementia were <1 (online supplemental material), indicating that stroke and dementia are diagnosed earlier among these individuals.

### Subgroup analyses

We tested subgroups by sex, age group and scan modality. The aHR between stroke and WMH was higher in males than females (p_interaction_<0.001), and the aHRs between stroke and both cortical infarcts and cerebral atrophy were higher in females than males (p_interaction_<0.01 and p_interaction_<0.001). The aHRs for WMH, lacunes, atrophy (all p_interaction_<0.001) and cortical infarcts (p_interaction_<0.05) were stronger in younger than older people, and aHRs for lacunes (p_interaction_<0.01) and cerebral atrophy (p_interaction_<0.001) were stronger with MRI than CT imaging. For dementia, aHRs for cerebral atrophy (p_interaction_<0.01) and WMH (p_interaction_<0.05) were stronger in males than females; aHRs for WMH, cerebral atrophy (all p_interaction_<0.001) and cortical infarcts (p_interaction_<0.01) were stronger in younger than older people and aHRs for cerebral atrophy were stronger for MRI than CT (p_interaction_<0.001) (online supplemental material).

## Discussion

This cohort study of 367 988 people demonstrates that those with clinically reported CCD or cerebral atrophy have a higher incidence of future stroke and dementia diagnosis. Compared with people with normal scans, those with cortical infarcts had the highest aHR of ischaemic stroke, and those with lacunes had the highest aHR of haemorrhagic stroke. Those with cerebral atrophy had the highest aHR of Alzheimer’s disease, and those with WMH had the highest aHR of vascular dementia. Compared with the general population (and those with normal scans), the 5-year absolute risk of dementia and stroke was substantially elevated in people with these scan reports, particularly dementia risk among those with cerebral atrophy and stroke risk among those with lacunes and cortical infarcts (online supplemental material).

As expected, the prevalence of CCD and atrophy phenotypes in this clinical cohort was generally higher than in research cohorts. For example, around 22% of the sample had a WMH at scan, compared with ~10% in research cohort studies but in line with ~28% in previous clinical cohort studies.^1 19^ In contrast, the prevalence of brain infarcts was similar to previous population research cohorts, ~10% compared with 10% (lacunes and cortical infarcts) in our study.^20^ The aHRs of WMH and deep brain infarct with stroke and dementia in research cohort studies were also stronger (aHR >2) than the maximally adjusted estimates from the present study.^4^ However, the aHRS reported here are similar to clinical studies for both stroke (aHRs 1.07–1.45)^11^ and dementia (aHRs 1.28–1.93).^10^ Lower aHRs in clinical than research cohort studies could be due to lack of precision in the measurement of brain imaging phenotypes in clinical compared with research studies or higher risk of death in people in clinical studies, hence a lower measured disease incidence. Overall, the observed aHRs between cortical infarcts and all stroke risk (1.8) and between cerebral atrophy and all dementia risk (1.7) are concordant with previous studies in non-stroke and poststroke populations, consistent with the view that stroke involves more discrete vascular lesions while dementia involves more brain tissue loss.

Our study had several strengths. First, a validated rules-based NLP pipeline was used to detect markers of CCD and atrophy in clinical radiology reports, and these markers were associated with future health over a long follow-up time. As CCD is difficult to identify with clinical coding, the application of NLP represents one of the most comprehensive methods of identifying CCD across a population. Text radiology reports are ideally suited for high-performance NLP due to their replicable structure, limited phenotypes and short length. Previous studies have not measured multiple phenotypes, have used smaller cohorts (Ns of 261 960 and 241 050) and have only examined older people.^10 11^ Second, we took a whole clinical population approach, including both academic centres, where almost all research is currently done, and district hospitals, which do not usually contribute to research. Very few studies of imaging phenotypes have sample sizes of more than a thousand, and one of the largest cohorts, UK Biobank, is aiming for imaging 100 000 participants.^21^ The large sample in the present study had sufficient statistical power to study subgroups and rare conditions such as Parkinson’s disease. However, the combination of less frequent phenotypes and rare conditions—such as cortical infarcts and Parkinson’s disease risk—limits statistical power and warrants studies in larger populations. Third, linking participants to health systems data allowed the ascertainment of multiple outcomes at low cost.^22^,^24^ Fourth, we included an ageing-related non-brain condition (colorectal cancer) that was not associated with CCD or atrophy, increasing confidence that the findings were not entirely due to residual confounding.

Our study had several limitations. First, rules-based NLP models are not state-of-the-art in the field relative to large language models (LLM). However, rules-based models tend to perform well,^25^ and the time taken for data governance approvals and limited compute resource meant that an LLM approach was not feasible when the project started. Second, our population was clinical; hence, imaging was only performed in people at higher risk; this selection bias could limit generalisability to research cohorts or asymptomatic screening. Furthermore, imaging request information was too brief to meaningfully classify reports by indication; therefore, stroke or dementia as a reason for the imaging request could not be entirely excluded. However, we excluded people with a diagnosis of stroke or dementia prior to, or within 6 months of, scanning and, in sensitivity analysis, found that extending this exclusion to 1 and 5 years made little qualitative difference to the pattern of aHRs. Third, we were only able to link to hospital, prescription and death data and did not have access to data from primary care, so some chronic conditions will have been underascertained. This also meant that important confounders, such as obesity or smoking, were not available, and residual confounding could explain some of our findings. Similarly, we could not account for prior interventions such as cardiac procedures that have been associated with increased risk of brain infarcts.^26^ Fourth, there may have been diagnosis inclusion bias for dementia subtypes, because evidence of CCD could have increased the chance of a clinical diagnosis of vascular rather than other dementia subtypes. Fifth, epilepsy was included as a control condition, but it has been associated with the presence of white matter lesions.^27^ Lastly, our imaging phenotypes were relatively crude and did not include quantification or location. Research studies have indicated that risks may differ with brain infarct subtype, location and size.^28^ However, UK clinical brain imaging reports generally do not have sufficient detail to estimate the severity of WMH or atrophy or the precise location of deep or cortical infarcts. The appearance of old deep infarcts (‘lacunes’) is similar to perivascular spaces, recent small subcortical infarcts, small subcortical haemorrhage or end-stage cavitation in a white matter hyperintensity and is often difficult to distinguish in a clinical image. Ongoing work analysing the images in this dataset will provide quantitative phenotyping.

We have demonstrated that NLP can uncover previously difficult-to-discover phenotypes in electronic health records at a scale and speed not previously possible. However, care is needed, as NLP can reflect systematic biases in the clinical text, for example, incompleteness in reports of acute illness and trends in language used in reports over time.^29^,^33^ NLP phenotypes could be used to identify non-coded outcomes in large cohort studies that rely on the electronic health record for outcome ascertainment or to identify populations eligible for clinical trials.^34^ This may be true, particularly for asymptomatic, incidentally discovered findings such as CCD.

This study emphasises that CCD or atrophy discovered during the process of routine healthcare is a risk marker for stroke and dementia. In a clinical population, a report of atrophy indicates an increased risk of subsequent dementia but does not make a clinical diagnosis; hence, further assessment with cognitive testing may be needed. Apparently, asymptomatic cortical infarcts or lacunes indicate an increased risk of subsequent stroke, and in the absence of evidence to support specific interventions, careful application of primary vascular prevention—chiefly management of hypertension and high LDL cholesterol—should be the mainstay of treatment. Recent guidelines from the European Stroke Organisation state that the best practice to prevent stroke or dementia in people with CCD remains uncertain.^35^ Notably, the majority of disease outcomes occurred within 5 years of CCD being observed; this window may be a useful focus for clinical trials and intervention studies.

In conclusion, we implemented an NLP algorithm across a whole population and found that CCD and atrophy were associated with a higher risk of both dementia and stroke up to 12 years later, with important differences between CCD exposures. Clinical trials to reduce risk in people with CCD are warranted.

## Supplementary material

10.1136/jnnp-2025-337689 online supplemental file 1

## Data Availability

Data may be obtained from a third party and are not publicly available.
